# Speaking in Public Coping Scale (ECOFAP): content and response process validity evidence

**DOI:** 10.1590/2317-1782/20242023200en

**Published:** 2024-05-27

**Authors:** Anna Carolina Ferreira Marinho, Adriane Mesquita de Medeiros, Eduardo de Paula Lima, Letícia Caldas Teixeira

**Affiliations:** 1 Programa de Pós-graduação em Ciências Fonoaudiológicas, Universidade Federal de Minas Gerais – UFMG - Belo Horizonte (MG), Brasil.; 2 Departamento de Fonoaudiologia, Faculdade de Medicina, Universidade Federal de Minas Gerais – UFMG - Belo Horizonte (MG), Brasil.; 3 Corpo de Bombeiros Militar de Minas Gerais - Belo Horizonte (MG), Brasil.

**Keywords:** Speech, Validation Study, Adaptation Psychological, Surveys and Questionnaries, Self-Testing

## Abstract

**Purpose:**

To present the content and response process validity evidence of the Speaking in Public Coping of Scale (ECOFAP).

**Methods:**

A methodological study to develop and validate the instrument. It followed the instrument development method with theoretical, empirical, and analytical procedures, based on the validity criteria of the Standards for Educational and Psychological Testing (SEPT). The process of obtaining content validity evidence had two stages: 1) conceptual definition of the construct, based on theoretical precepts of speaking in public and the Motivational Theory of Coping (MTC); 2) developing items and response keys, structuring the instrument, assessment by a committee with 10 specialists, restructuring scale items, and developing the ECOFAP pilot version. Item representativity was analyzed through the item content validity index. The response process was conducted in a single stage with a convenience sample of 30 people with and without difficulties speaking in public, from the campus of a Brazilian university, belonging to various social and professional strata. In this process, the respondents’ verbal and nonverbal reactions were qualitatively analyzed.

**Results:**

The initial version of ECOFAP, consisting of 46 items, was evaluated by judges and later reformulated, resulting in a second version with 60 items. This second version was again submitted for expert analysis, and the content validity index per item was calculated. 18 items were excluded, resulting in a third version of 42 items. The validity evidence based on the response processes of the 42-item version was applied to a sample of 30 individuals, resulting in the rewriting of one item and the inclusion of six more items, resulting in the pilot version of ECOFAP with 48 items.

**Conclusion:**

ECOFAP pilot version has items with well-structured semantics and syntactic, representing strategies to cope with speaking in public.

## INTRODUCTION

Speaking in public is a form of oral communication in which the speaker faces an audience to share ideas, inform, entertain, or persuade a group of people^(1)^. Speaking well in public involves having both what to say and the communication skills to say it^(2-4)^. Many people feel uneasy or stressed to speak in public^(5)^_,_ reinforcing that this activity is one of the most prevalent fears in the world population^(6-8)^. On the other hand, it is known that how people cope with adversities either reduces or increases their vulnerability to stress, interfering with their health and well-being^(9,10)^.

The set of strategies people use to adapt to and cope with stressful situations is named coping^(11)^. One coping theory model that traditionally stands out is that by Lazarus and Folkman^(11)^, which is used as a theoretical reference in many studies^(12,13)^. It encompasses two categories that can be used alone or in combination to cope with the stressful situation: problem-focused coping (strategies aimed at the situation that originated the stressful event) and emotion-focused coping (regulatory strategies to change the person’s emotional response to a stressful situation)^(10)^.

Other coping models were presented based on the study by Lazarus and Folkman^(11)^, including the Motivational Theory of Coping (MTC)^(10,14-16)^. In MTC, coping responses are approached as self-regulatory actions developed from behavior patterns, associated with the temperamental characteristics, bond quality, and the context to which the person belongs^(9,10,14-16)^.

MTC organizes coping in a three-level hierarchical structure model: 1) lower level (coping responses); 2) intermediate level (coping strategies); and 3) upper level (with 12 coping categories/families: “solving problems”, “seeking information”, “helplessness”, “avoidance”, “self-confidence”, “seeking support”, “delegating”, “isolation”, “accommodation”, “negotiation”, “submission”, “opposition”. Each one of these coping categories/families is related to a type of cognitive appraisal of the stressful event, a basic need, and an adaptive process^(9,10)^.

The cognitive appraisal of the stressful event may be classified as a challenge (when the person believes they can cope with the stressful event) or as a threat (when they perceive the situation as psychological damage)^(10,11)^.

MTC basic needs may be related to competence, bonding, and autonomy. Competence refers to the human desire to achieve goals and be effective in social interactions, bonding refers to the process of establishing close interpersonal relationships and creating reliable ties to feel valued, and autonomy refers to the freedom to make choices as one interacts with the environment^(9,10,14-16)^.

Lastly, three adaptive processes are defined as personal interpretations of potentially stressful situations. The self-referential adaptive process of coordinating actions and contingencies in the environment is related to the psychological need for competence. The adaptive process of coordinating confidence and social resources available is related to the need for bonding. The adaptive process of coordinating preferences and options available to make choices is related to the basic human psychological need for autonomy^(9-11)^.

Strategies to cope with speaking in public can help the person address better the stressful situation^(1,3,17,18)^. Some examples of such strategies include studying the content that will be presented, training to speak in public with other people, seeking support from peers, or investing in communication advisory^(1-3,17-19)^. Even though the literature indicates these and other strategies, no instrument was found to self-assess strategies to cope with speaking in public^(20)^. There are few existing self-perception instruments, including the Self-Statements During Public Speaking – SSPS^(5)^, which assesses the cognitive dimension of public speaking, the Public Speaking Anxiety Scale – PSAS^(6)^, which measures the behavioral, cognitive and physiological components of anxiety when speaking in public and the Personal Report of Public Speaking Apprehension –PRPSA^(20)^ which assesses the level of discomfort or nervousness that individuals experience when facing speaking situations in front of an audience, including the fear of being judged.

Therefore, this study proposes a coping scale to assess speaking in public, named Speaking in Public Coping Scale (in Portuguese, *Escala* Coping *para Fala em Público* [ECOFAP]). It was developed based on the Speaking in Public and MTC theoretical framework^(9,10,14-16)^. This theoretical model was chosen because of the MTC coverage of self-regulation mechanisms, as this construct is influenced by specific genetic, physiological, and social life-cycle processes.

The development of ECOFAP followed the epidemiological methodological steps necessary for its scientific robustness and validity. All instrument validation and reliability stages were followed according to the Standards for Educational and Psychological Testing (SEPT), which is a manual that guides test development and validation, requiring five pieces of validity evidence: test content; response processes; internal structure; relationship with other variables; and evidence based on the consequences of the tests^(21)^.

This article will present the validity evidence of content and response process^(22,23)^. Content validity is related to the format of questions/items, including their syntactic and semantic structure^(23-26)^ and the necessary procedures to score them^(25,26)^. The response process is the application of the instrument (which has been reformulated after consensus among expert judges) to different strata of the target population to verify the respondents’ performance.

Given the above, this study aimed to present ECOFAP validity evidence based on its content and response process.

## METHOD

This is a methodological instrument development and validation study. It followed the instrument development model proposed by Luíz Pasquali^(24,25)^, which encompasses theoretical, empirical, and analytical procedures, meeting the validity criteria pointed out by the Standards for Educational and Psychological Testing (SEPT)^(21)^. The study was approved by the Research Ethics Committee under evaluation report no. 5.735.670/2022.

The theoretical construct of ECOFAP was developed based on the theoretical framework of speaking in public and MTC^(9,10,14-16)^. Content validity evidence was obtained in two stages:1) item development method and 2) expert judges’ assessment method, and response process validity evidence was obtained in a single stage: 1) Response validation in individuals with and without difficulties speaking in public.

### Content validity evidence

1. First stage: Item development method

- **Item development sources:**

- Verifying the integrative literature review of instruments to assess speaking in public, by Marinho et al.^(20)^.
- Consolidating on the speaking in public construct, grounded on the authors’ more than 10 years of clinical experience.
- Aligning items according to the conceptual bases of the MTC structural model^(9,10,14-16)^.
- Interviewing people with difficulties in speaking in public undergoing speech-language-hearing treatment to improve communication at a teaching clinic of a Brazilian public university.
- Defining the syntactic and semantics of the items, according to Pasquali’s 10 criteria^(25)^.
  1. Behavioral criterion: the item must express a behavior.
  2. Objectivity: easily identifiable answers.
  3. Simplicity: the item must convey a single idea.
  4. Clarity: being understandable to all strata of the target population and avoiding long sentences.
  5. Relevance: the sentence/expression must be able to assess the construct in question.
  6. Precision: each item must be different from the other ones.
  7. Variety: using diversified language – using the same terms may lead people to mistake sentences, rather than differing them. For instance, develop half the sentences as affirmative ones and the other half as negative ones.
  8. Modality: avoiding extreme expressions, such as “awful” or “excellent”.
  9. Typicity: sentences with expressions typical of the attribute.
  10. Credibility: the item must not seem incoherent, pointless, or inappropriate to the age group for which it was developed.

The number of items in the construct followed the guidance proposed by Pasquali^(25)^, which recommends that an instrument must have at least twenty items. The initial verbs of the ECOFAP items were formulated to represent an action/effort verbs. Each of the twelve coping families should have at least two items.

1. Second stage: Expert judges’ assessment method

The first ECOFAP version, made with 46 items,was analyzed by a committee of 10 expert judges (five speech-language-hearing therapists and five psychologists), following the Delphi method premises, which aim to gather and systematize, by consensus, the opinions of specialists on the topic^(26-28)^.

The judges were from various Brazilian states. The five speech-language-hearing therapists were voice specialists, with a doctoral degree and more than 5 years of clinical experience in occupational voice use. The five psychologists had a doctoral degree and were researchers with experience in the coping construct.

They were recruited between December 2021 and June 2022 with an invitation letter sent via e-mail, which also had a link to the form in Google Forms, an informed consent form, the objectives of the study, and instructions to analyze the material and judge each item proposed by the researchers.

### Steps to the judges’ assessment

1. Assessing each item in terms of theoretical appropriateness (whether its content represents the construct it is meant to measure), to which they could answer with 3 of adequate, 2 of undecided, or 1 of inadequate.
2. Assessing each item in terms of textual pertinence (semantic adequacy, vocabulary – whether the item expresses only the idea it is meant to assess), to which they could answer with 3 of adequate, 2 of undecided, or 1 of inadequate.
3. Justifying the assessment of each item judged as inadequate and suggesting changes to it.
4. Classifying items into each of the 12 MTC coping families: “solving problems”, “seeking information”, “helplessness”, “avoidance”, “self-confidence”, “seeking support”, “delegating”, “isolation”, “accommodation”, “negotiation”, “submission”, “opposition”. The items were randomized to avoid selection bias.
5. Indicating whether the item could represent more than one coping family. If so, the judge should inform which ones it would represent.
6. After the evaluators had presented their suggestions and comments, the researchers made the necessary changes in ECOFAP and organized its second version, which was again submitted to the judges’ appraisal, following the same assessment steps. The second version was sent with 60 items.

After the judges’ second assessment of ECOFAP, its second version was readjusted based on their comments and quantitatively assessed. The protocol items were allocated in a database and their item content validity index (I-CVI) was calculated, analyzing the percentage of expert judges who agreed with each instrument item. Only values above 0.78 were accepted, following Polit et al^(29)^. theoretical concepts. This analysis was performed in SPSS statistical software, version 25.0. 18 items were excluded. After the qualitative and quantitative analyses, the authors developed the third ECOFAP version, which they named the pilot version. It had 42 items and was assessed by individuals with and without difficulties speaking in public in the response process validation stage.

### Response process validity evidence

This evidence process verifies whether the item development has flaws or poses comprehension difficulties and whether the instrument questions are adequate for various population strata. This process had a single stage:

1. Response validation stage with individuals with and without difficulties speaking in public

The ECOFAP third version, with 42 items, was assessed by a convenience sample, represented in table 1. The sample was composed of 30 people, between 18 and 40 years old, attending a Brazilian university. The majority of participants were female (66.6%), with incomplete higher education (53.2%), self-reported having difficulties speaking in public (73.3%). Regarding the profession, the majority were university students (43.3%), followed by secretaries and administrative technicians (24.7%), university professors (21.5%) and doormen (10.7%).

**Table 1:** Sociodemographic characteristics and self-reported difficulties in public speaking (n=30)

**Variables**	**N**	**%**
**Gender**		
Female	20	66.6
Male	10	33.4
**Age group**		
18-22 years	3	10.2
23-26 years	12	43.1
27-31 years	9	26.4
32-40 years	6	20.3
**Education**		
High school	9	26.6
Incomplete higher	18	53.2
Graduated	6	20.2
**Self-reported having difficulties speaking in public**		
Yes	22	73.3
No	8	26.7
Professions		
Doormen	3	10.7
University students	12	43.1
University professor	6	21.5
Secretaries and administrative technicians	9	24.7

Caption: N = number of cases, % = frequency

The collection took place in July 2022. The sample comprised literate individuals of both sexes above 18 years old. All participants signed an informed consent form with the study objectives and judgment instructions for each item proposed by the researchers. All interviews were held by the same researchers to control possible biases. To control possible biases, all interviews were carried out by two speech therapist volunteers. Before applying the instrument, the researchers provided training to the volunteer speech therapists, explaining the objective of this validation stage, and providing guidance on the detection of operational difficulties, such as time of application of the instrument, non-verbal reactions of the interviewees (facial expressions, doubts, impatience, manifestations of anxiety and body language) and suggestions from participants. Such characteristics should be recorded by volunteer speech therapists immediately after applying each question qualitatively, without using scores.

After participants answered each question, on a Likert scale from 0 (totally disagree) to 5 (totally agree), the evaluator asked, “Did you understand the question?”, to verify whether the item was clear, and asked them to repeat the question as they had understood it, *“Could you, please, repeat the question as you understood it?”*. When their answer had a different element from the original question, their answer was transcribed during the interview. This paraphrasing strategy helped analyze their comprehension of what they were being asked, enabling changes.

In the process, researchers verified the instrument administration time, difficulties understanding the items, and interviewees’ nonverbal reactions (facial expressions, questions, impatience, anxiety, and body language).

After administering ECOFAP, the researchers met and qualitatively assessed each item regarding the respondents’ comprehension and applicability, classifying the items as adequate (when no adjustments were needed) or inadequate (when the item had not been satisfactorily understood). The authors of ECOFAP made the changes they deemed necessary by consensus, developing a new ECOFAP version.

## RESULTS

The results present ECOFAP content and response process validity evidence.

Chart 1 presents 24 assessment items proposed by the researchers, categorized into the six coping families related to the cognitive assessment of the stressful situation as a challenge, along with the comments of the committee of specialists on each item. Items 1, 3, 4, 5, 9, 10, and 16 were considered adequate by the committee of specialists. Items 2, 7, 8, 13, 17, and 18 were also considered adequate, but the judges suggested some syntactic changes. Items 6, 11, 12, 14, 15, 19-24 were considered inadequate, not representing any coping strategy.

**Chart 1:** Assessment items of the first ECOFAP version related to the cognitive assessment of speaking in public as a challenge (n = 24)

**Coping families**	**First ECOFAP version**	**Comments about the first ECOFAP version**
**Solving problems**: Active attempt to change the situation or its consequences. Planning strategies, and instrumental actions.	1-I List the main topics of what I’ll say	-
	2-I outline a connection between my ideas	Replace “outline” with “make”, “establish”
	3-I train what I’ll say	-
	4-I plan my presentation to speak better	-
	5-I try to speak clearly	-
	6-I keep my voice firm	This item does not represent a coping strategy
**Seeking information:** Active attempt to learn more about the situation. Reading, studying, asking others.	7-I try to learn more from public speaking advisory	“Advisory” may not be a popular term for most people
	8-I participate in oratory courses	“Participate” as a student
	9-I read to learn more about how to speak in public	-
**Self-confidence**: Active attempt to diminish emotional anguish. Behavioral regulation and positive self-talk.	10-I keep a confident body posture	-
	11-I believe much of my nervousness is not visible	This item does not represent a coping strategy
	12-I avoid negative thoughts about myself	This item does not represent a coping strategy
	13-I try to remain calm	Complete the sentence with: “when I speak”
	14-I try to see it as an opportunity to grow	This item does not represent a coping strategy
**Seeking support:** Active attempt to use social resources available to cope with the stressful situation by seeking contact, help, and social references.	15-I hear my peers’ opinions	This item does not represent a coping strategy
	16-I seek professional support	-
	17-I practice in front of familiar people	Complete: “I practice my speech”
	18-I talk to more experienced people	Reformulate this item
**Accommodation:** Active attempt to redirect attention away from the stressful situation, focusing on pleasant activities or seeing the situation from a different perspective. Cognitive restructuring, acceptance, minimization.	19-I’m afraid of speaking, but I go on	This item does not represent a coping strategy
	20-I feel my heart beating very fast, but I believe it’ll go away soon	This item does not represent a coping strategy
	21-I learn good things in every new experience	This item does not represent a coping strategy
**Negotiation:** Active attempt to find new options. Developing solutions that balance individual priorities with restrictions imposed by the situation and establishing new goals. Exchange.	22-I set small goals every day	This item does not represent a coping strategy
	23-I prepare answers to questions I suppose I’ll be asked	This item does not represent a coping strategy
	24-Instead of getting worried, I imagine I’ll do a good job	This item does not represent a coping strategy

Presentation form adapted from Vasconcelos and Nascimento^(30)^, Skinner and Zimmer-Gembeck^(16)^ and Skinner et al.^(9)^

Chart 2 shows the other 22 assessment items of the first ECOFAP version, categorized in the six coping families related to the assessment of speaking in public as a threat, along with the comments of the committee of specialists regarding each item. The judges assessed items 36, 37, 38, and 40 as adequate. Assessment items 25, 27, and 41 were also considered adequate, but they suggested changes. Items 26 to 35, 39, and 42 to 46 were considered inadequate because they did not represent any coping strategy.

**Chart 2:** Assessment items of the first ECOFAP version related to the cognitive assessment of speaking in public as a threat (n = 22)

**Coping families**	**First ECOFAP version**	**Comments about the first ECOFAP version**
**Helplessness**: The person feels powerless in the situation. Passivity, hesitation, discouragement.	25-I think I’ll forget the words	Replace “I think” with “I’m afraid”
	26-I feel I can’t calm down	This item does not represent a coping strategy
	27-I freeze when I have to speak	“I freeze, I get stuck”
**Avoidance:** Active attempt to escape the situation and the difficulties related to it. Pessimism, mental distancing.	28-I speak faster to get rid of the situation	Specify the situation of speaking in public
	29-I avoid looking at people while I speak	This item does not represent a coping strategy
	30-I wish my speech would end soon	This item does not represent a coping strategy
	31-I believe I’m not objective when I speak	This item does not represent a coping strategy
**Delegation:** The person feels sorry for themselves, figuring they do not have enough resources to cope with the stressful event. Complaint, self-guilt, groaning.	32-I don’t know where to put my hands while I speak	This item does not represent a coping strategy
	33-I believe no one pays attention when I’m speaking	This item does not represent a coping strategy
	34-I imagine no one understands what I say	This item does not represent a coping strategy
	35-I consider speaking in public a suffering	This item does not represent a coping strategy
**Isolation:** Active attempt to avoid people or not letting them know how they feel about the situation. Social distancing, avoiding others, loneliness.	36-I avoid places where I have to speak	-
	37-I avoid giving my opinion, even when I have something to say	-
	38-I hide what I feel from my peers when I have to speak	-
	39-I look away from listeners	This item does not represent a coping strategy
	40-Ia void talking to peers about my difficulties in speaking in public	-
**Submission:** Active attempt to keep passively and repetitively focused on negative and harmful aspects of the situation. Stiffness, rumination, intrusive thoughts.	41-I believe I’ll be a failure	Complete the idea of the item
	42-I think that if I make a mistake, I’ll not be able to resume my speech	This item does not represent a coping strategy
	43-I think I’ll not be able to answer questions	This item does not represent a coping strategy
**Opposition:** Active attempt to use angry behavior, bursts, to remove obstacles imposed by the stressful situation. Aggressiveness, explosion, blaming others.	44-I feel no one empathizes with me	This item does not represent a coping strategy
	45-I get tired from repeating what I said	This item does not represent a coping strategy
	46-I believe no one pays attention when I speak	This item does not represent a coping strategy

Presentation form adapted from Vasconcelos and Nascimento^(30)^, Skinner and Zimmer-Gembeck^(16)^ and Skinner et al.^(9)^

Tables 2 and 3 show the second ECOFAP version with 60 items. Table 3 presents 30 items rewritten based on the expert judge’s committee suggestions, classified in the six coping families related to assessing the stressful situation as a challenge, along with the comments of the second appraisal round and I-CVI. Items 2, 3, 4, 6, 7, 9, 10, 11, 12, 18, 19, 21, 22, 23, 26, 27, 29, and 30 were well-assessed and had representative I-CVI, while items 1, 5, 8, 13, 16, 17, 20, 24, 25, and 28 were considered inadequate and removed.

**Table 2:** Assessment items of the second ECOFAP version, categorized in coping families related to assessing the situation as a challenge, along with comments and item content validity index (n = 30)

**Coping families**	**Second ECOFAP version**	**Comments about the second ECOFAP version**	**I-CVI**
**Solving problems**	1-I study the content about which I’m going to speak	This item represents the family “seeking information”	0.64
	2-I previously organize my ideas	-	0.90*
	3-I train aloud what I’m going to say	-	0.90*
	4-I prepare a visually attractive presentation	-	0.85*
	5-I focus on the content of my presentation while I’m speaking	This item does not represent a coping strategy	0.70
	6-I strive to pronounce my words clearly	-	0.89*
**Seeking information**	7-I read about how to speak in public	-	0.90*
	8-I train in how to speak in public	This item represents other families: Seeking support, Solving problems	0.60
	9-I pay attention to how good speakers speak	-	0.82*
	10-I pay attention to how peers act in this situation	-	0.85*
	11-I watch videos on how to speak well	-	0.89*
**Self-confidence**	12-I try to keep a confident body posture	-	0.92*
	13-I tell myself this is a good opportunity to learn	Remove item	0.30
	14-I focus on positive thoughts	-	0.80*
	15-I try to keep calm while I speak	-	0.95*
	16-I try to see the situation as an opportunity to grow	This item does not represent a coping strategy	0.50
**Seeking support**	17-I ask peers and friends to help me	This item does not represent a coping strategy	0.55
	18-I practice my speech with familiar people	-	0.80*
	19-I seek professional support	Complete with “to learn to speak well in public”	0.85*
	20-I hear my peers’ opinions	This item does not represent a coping strategy	0.60
	21-I ask more experienced peers what they do to speak well	-	0.80*
**Accommodation**	22-I walk around	Complete with “to distract the mind before a public presentation”	0.80*
	23-I watch television	Complete with “to distract from my concern with my presentation”	0.95*
	24-I do some type of relaxation or meditation	Remove item	0.20
	25-I do breathing exercises	This item does not represent a coping strategy	0.50
	26-I listen to music	Complete with “to relax before speaking in public”	0.80*
**Negotiation**	27-I set small goals every day	Complete with “to be a good speaker”	0.90*
	28- I prepare answers to questions I suppose I’ll be asked	This item does not represent a coping strategy	0.45
	29-I try to replace the task of speaking with another assignment	-	0.80*
	30-I try to get more time to prepare myself	-	0.95*

Caption: I-CVI = Item content validity index

* I-CVI > 0.78

**Table 3:** Assessment items of the second ECOFAP version categorized in the coping families related to assessing the situation as a threat, along with comments and item content validity index (n = 30)

**Coping families**	**Second ECOFAP version**	**Comments about the second ECOFAP version**	**I-CVI**
**Avoidance**	31-I suggest someone else to speak in my place	“I avoid this task and suggest someone else to speak in my place”	0.80*
	32-I postpone situations of speaking in public	-	0.90*
	33-I speak faster to finish speaking sooner	-	0.95*
	34-I come up with an excuse not to speak in public	-	0.85*
	35-I change subjects when someone asks me a question	This item does not represent a coping strategy	0.40
**Delegation**	36-I complain to my peers when I’m the one who has to speak	-	0.90*
	37-I blame myself for not wanting to speak	-	0.85*
	38-I complain to my family that I have to go through this situation	Remove item	0.60
	39-I complain that I can’t speak well	Remove item	0.55
	40-I keep hoping someone will go through the situation in my place	-	0.85*
**Helplessness**	41-I get discouraged when I think about having to speak in public	Replace “I get discouraged” with “It discourages me”	0.90*
	42-I freeze when I have to speak in public	“I imagine myself freezing”	0.80*
	43-I imagine negative results	This item does not represent a coping strategy	0.40
	44-I try to speak better but I can’t	-	0.95*
	45-I reflect on what I’m going to say but I find no way out	-	0.80*
**Isolation**	46-I look away from listeners	This item does not represent a coping strategy	0.45
	47-I avoid giving my opinion, even when I have something to say	-	0.90*
	48-I avoid places where I have to speak	-	0.85*
	49-I avoid talking to my peers about my difficulties in speaking in public	-	0.84*
	50-I hide from my peers how I feel when I have to speak	-	0.92*
**Submission**	51-I get stuck to the idea that there is no use in getting prepared	Replace “I get stuck to” with “I insist on”	0.85*
	52-I insist on the idea that I’ll not be successful	-	0.90*
	53-I get prepared for the worst	This item does not represent a coping strategy	0.30
	54-I tell myself that it is tough to cope with this situation	-	0.85*
	55-I keep ruminating about negative sensations caused by the situation	This item does not represent a coping strategy	0.25
**Opposition**	56-I reply harshly when someone interrupts me	-	0.84*
	57-I blame others when I have to speak	-	0.90*
	58-I get irritable when someone disagrees with what I said	-	0.85*
	59-I take my stand rudely	This item does not represent a coping strategy	0.35
	60-I get impatient when others ask me to repeat what I said	Replace “I get impatient” with “I react impatiently”	0.95*

Caption: I-CVI: Item content validity index

* I-CVI > 0.78

Table 3 presents the other 30 items of the second ECOFAP version, rewritten based on the expert judge’s committee suggestions, classified in the six coping families related to assessing the stressful situation as a threat, along with the comments of the second appraisal round and I-CVI. The judges classified items 31 to 34, 36, 37, 41, 42, 44, 45, 47, 48, 49, 50, 51, 52, 54, 56, 57, 58, and 60 as adequate, with representative I-CVI. Items 35, 38, 39, 43, 46, 53, 55, and 59 were considered inadequate, with a low I-CVI.

Chart 3 shows the ECOFAP pilot version with 48 items. This version was developed based on restructuring the second ECOFAP version and administrating it to the 30-people convenience sample described in the method of this article to obtain response process validity evidence.

**Chart 3:** Assessment items of the ECOFAP pilot version in the coping families (n = 48)

**Coping families**	**ECOFAP pilot version**
**CHALLENGE**	
**Solving problems**	1-I previously organize my ideas
	2-I practice aloud what I’ll say
	3-I prepare a visually attractive presentation
	4-I strive to pronounce my words clearly
**Seeking information**	5-I read about how to speak well in public
	6-I pay attention to how good speakers speak
	7-I watch videos about how to speak well
	8-I pay attention to how peers act in this situation
**Self-confidence**	9-I see myself speaking well
	10-I try to keep a confident body posture
	11-I try to keep calm when I speak
	12-I focus on positive thoughts
**Seeking support**	13-I seek professional support to learn to speak well in public
	14-I ask more experienced peers what they do to speak well
	15-I practice my speech with familiar people
	16-I seek suggestions from my peers on how they cope with the situation
**Accommodation**	17-I listen to music to relax before speaking in public
	18-I walk around to distract my mind before a public presentation
	19-I watch television to distract from my concerns with the presentation
	20-I focus on something good that may result from this situation
**Negotiation**	21-I set small goals every day to become a good speaker
	22-I try to have more time to get prepared
	23-I make it clear to everyone that I do not do this task well
	24-I try to replace the task of speaking with another assignment
**THREAT**	
**Helplessness**	25-Thinking about speaking in public discourages me
	26-I imagine myself freezing when I start speaking in public
	27-I try to speak better but I can’t
	28-I reflect on what I’ll say but I find no way out
**Avoidance**	29-I postpone the situation of speaking in public
	30-I come up with an excuse not to speak in public
	31-I speak faster to finish speaking sooner
	32-I avoid this activity and suggest someone else to speak in my place
**Delegation**	33-I complain to peers that I’m the one who has to speak
	34-I blame myself for not wanting to speak in public
	35-I ask someone to speak in my place
	36-I reprehend myself for not speaking well
**Isolation**	37-I avoid talking to my peers about my difficulty
	38-I hide from my peers how I feel when I have to speak
	39-I avoid places where I have to speak
	40-I avoid giving my opinion in public, even when I have something to say
**Submission**	41-I insist on the idea that there is no use in getting prepared
	42-I insist on the idea that I’ll not be successful
	43-I tell myself that it is tough to cope with this situation
	44-I tell myself that my presentation will go wrong
**Opposition**	45-I reply harshly when someone interrupts me
	46-I get irritable when someone disagrees with what I’m saying
	47-I react impatiently when others ask me to repeat what I said
	48-I blame others for having to speak

After administering it, the researchers assessed each item by consensus, categorizing as adequate the ones that did not need any adjustment and inadequate the ones that were not satisfactorily understood. The item “I read about how to speak in public” was rewritten as “I read about how to speak well in public”. Items “I see myself speaking well”, “I seek suggestions with my peers about how to cope with the situation”, “I focus on something good that may result from this situation”, “I make it clear to everyone that I do not this task well”, “I ask someone to speak in my place”, and, “I tell myself that my presentation will go wrong” were added based on the participants’ opinions.

## DISCUSSION

The results show the ECOFAP content and response process validity evidence. Such evidence was essential to adjust the theoretical, contextual, semantic, and syntactic aspects of the initial ECOFAP versions. The authors recommend that the results of the content and response process validity stages be publicized before continuing the investigation of the subsequent instrument validity evidence stages, ensuring the methodological rigor necessary to construct instruments^(21)^.

ECOFAP is a self-assessment scale on strategies to cope with speaking in public. Self-assessment instruments are known to help people ponder about certain aspects often not spontaneously reported and influence their readiness to change regarding the issue^(31,32)^. Self-assessments also help verify the person’s perception of their self-regulatory capacity^(33)^. Authors argue that self-regulation is essential to the learning process, generalization of new skills, and long-term management or maintenance of acquired behaviors^(34)^.

ECOFAP development was based on MTC, by Skinner et al.^(9,10,14-16)^, who understand coping as a self-regulatory action to monitor response behaviors to stressful situations^(9,10,14-16,30)^. Other studies in health also used MTC as the basis to develop coping instruments^(35,36)^.

The first ECOFAP version was developed with 46 items, divided into the families and their respective adaptive processes, as shown in Charts 1 and 2. The items were randomized for the judges’ appraisal to avoid analysis bias^(36)^. This methodological precaution aims to prevent judges from the tendency to assess all items the same way, not considering their content or format differences. When items are randomized, each one is assessed independently, ensuring a more precise and reliable analysis, and avoiding the evaluator’s fatigue, as item presentation is not repetitive^(36)^. The qualitative assessment of items showed that most suggestions referred to the use of language, verbs, and coping families. The language knowingly needs to be clear, and syntactic and semantic aspects of the sentence must ensure cohesion and present a single, understandable idea aimed at the target population^(23-25)^.

In this regard, some terms had to be revised, such as replacing “advisory” with training or courses. In the first version, 20 items were assessed as inadequate because they represented a feeling, rather than a strategy to cope with speaking in public. The verbs in these items had to be revised to characterize them as a regulatory action to adjust and cope with stressful situations.

Studies describe difficulties in developing instrument items and classifying them in the 12 MTC coping families^(37)^. Developing items is known to be a tough process, requiring methodological rigor and often needing the analysis of specialists not involved in the process – which reinforces the importance of this content validity phase. After the comments and suggestions of the first version, the authors met, discussed, and made new adjustments. Items were reformulated or removed, while other ones were included and reclassified in other families. All these changes were only possible thanks to the specialists’ comments, leading authors to restructure the items, as observed in other studies in the literature^(37,38)^.

The second ECOFAP version with 60 items (Tables 2 and 3) was better accepted by the judges, with small suggestions. Qualitative and quantitative assessments were carried out in this phase. The quantitative assessment is recommended to ground researchers’ decision-making concerning instrument adjustments, characterizing the agreement between judges through the analysis of the content validity index^(24-26)^. The results show that most items’ I-CVI had a high degree of agreement, demonstrating that the items were well-written and adequately followed the validation stages to this end.

The 48 items in the third ECOFAP version (Chart 3) were defined based on the analysis result of the content validity index, followed by the application of a pilot study in various target population strata and their opinions. Studies point out that investigating response process validity evidence complemented test content validity evidence and was highly relevant to adjusting the instrument based on verbal and nonverbal reactions of the target population^(23,38)^. In this phase, they suggested including five items and rewriting one. The changes were considered appropriate to meet the simplicity criteria proposed by Pasquali, adapting the language to the target population^(24,25)^.

The limitations of this study are related to the absence of studies on criterion validity (comparing with an external measure), construct validity (analyzing the factorial structure), reliability (temporal stability through test-retest and internal consistency), and the establishment of a cutoff score with clinical meaning.

The first, second, and pilot version presented here are not the final ECOFAP version. ECOFAP will still undergo internal structure evidence investigation to analyze its psychometric characteristics. Once the process of validating ECOFAP is finished, it will be available to be used in speech-language-hearing research and assistance as a public speaking self-perception and self-regulation instrument. It is believed that the scale will help conduct assistance and provide outcome measures for scientific research.

## CONCLUSION

ECOFAP is a self-assessment scale of strategies to cope with speaking in public, developed and validated with test content and response process evidence. Its items have adequate semantic and syntactic structures that represent the theoretical self-regulation construct of speaking in public. Researchers need to consider such evidence to use reliable instruments that are appropriate to the target population.
