# Supplementary material for: Speaking in Public Coping Scale (ECOFAP): content and response process validity evidence
Source: Codas. 2024 May 27;36(4):e20230200. doi: 10.1590/2317-1782/20242023200en (PMC11189152; doi:10.1590/2317-1782/20242023200en)
Supplement: Translation

# Translation: Escala de Coping para Fala em Público (ECOFAP): evidências de validade de conteúdo e processos de resposta

**DOI:** 10.1590/2317-1782/20242023200pt

**Authors:** Anna Carolina Ferreira Marinho, Adriane Mesquita de Medeiros, Eduardo de Paula Lima, Letícia Caldas Teixeira

**Affiliations:** 1 Programa de Pós-graduação em Ciências Fonoaudiológicas, Universidade Federal de Minas Gerais – UFMG - Belo Horizonte (MG), Brasil.; 2 Departamento de Fonoaudiologia, Faculdade de Medicina, Universidade Federal de Minas Gerais – UFMG - Belo Horizonte (MG), Brasil.; 3 Corpo de Bombeiros Militar de Minas Gerais - Belo Horizonte (MG), Brasil.

**Keywords:** Fala, Estudo de Validação, Adaptação Psicológica, Inquéritos e Questionários, Autoteste

## Abstract

**Objetivo:**

Apresentar as evidências de validade baseadas no conteúdo e nos processos de resposta da Escala de Coping para a Fala em Público (ECOFAP).

**Método:**

Estudo metodológico de elaboração e validação de instrumento. Seguiu-se o modelo de elaboração de instrumentos com procedimentos teóricos, empíricos e analíticos, baseados nos critérios de validade do Standards for Educational and Psychological Testing (SEPT). O processo de obtenção das evidências de validade baseadas no conteúdo foi realizado em duas etapas: 1) definição conceitual do construto, elaborado com base nos preceitos teóricos da fala em público e da Teoria Motivacional do Coping (TMC); 2) elaboração dos itens e chave de respostas, estruturação do instrumento, avaliação por comitê de dez especialistas, reestruturação dos itens da escala, realizada em três momentos, até a elaboração da versão piloto da ECOFAP. O processo de resposta foi realizado com amostra de conveniência de 30 indivíduos, com e sem dificuldades de fala em público, no campus de uma universidade brasileira, pertencentes a diferentes extratos sociais e profissões. Nesse processo, foram analisadas qualitativamente as reações verbais e não verbais dos respondentes.

**Resultados:**

A primeira versão da ECOFAP, composta por 46 itens, foi avaliada pelos juízes e posteriormente reformulada, resultando em uma segunda versão com 60 itens. Essa segunda versão foi novamente submetida à análise de especialistas e calculado o índice de validade de conteúdo por item. Foram excluídos 18 itens, originando uma terceira versão de 42 itens. As evidências de validade com base nos processos de resposta da versão de 42 itens foram aplicadas em uma amostra de 30 indivíduos, resultando na reescrita de um item e inclusão de mais seis itens, originando a versão piloto da ECOFAP de 48 itens.

**Conclusão:**

A versão piloto da ECOFAP apresenta itens bem estruturados semântica e sintaticamente que representam estratégias de enfrentamento para a fala em público.

## INTRODUÇÃO

Falar em público é uma forma de comunicação oral, na qual o falante fica face a face com uma audiência com a intenção de compartilhar ideias, informar, entreter ou persuadir um grupo de pessoas^(1)^_._ Falar bem em público envolve ter o que dizer e a habilidade comunicativa de saber o como dizer^(2-4)^. Muitos oradores, diante de situações de fala em público, se sentem desconfortáveis ou ficam estressados^(5)^_,_ reforçando o quanto essa atividade é um dos temores mais prevalentes entre a população mundial^(6-8)^. Em contrapartida, sabe-se que a maneira como a pessoa lida com as adversidades reduz ou aumenta a vulnerabilidade ao estresse, interferindo na saúde e no bem-estar^(9,10)^.

O conjunto de estratégias que o indivíduo utiliza como resposta para se adaptar e enfrentar situações estressoras é denominado *coping* ^(11)^. Entre os modelos teóricos de *coping,* destaca-se tradicionalmente o modelo de Lazarus e Folkman^(11)^, utilizado como referencial teórico por muitos estudos^(12,13)^. Esse modelo compreende duas categorias: *coping* focado no problema (estratégias que visam atuar na situação que originou o evento estressor) e *coping* focado na emoção (estratégias regulatórias para modificar a resposta emocional do indivíduo em relação à situação estressora) e que podem ser utilizadas isolada ou simultaneamente, para enfrentamento da situação estressora^(10)^.

A partir do trabalho de Lazarus e Folkman^(11)^ surgiram outros modelos de *Coping,* entre eles, destaca-se a Teoria Motivacional do *Coping* (TMC)^(10,14-16)^. Na TMC as respostas de *coping* são compreendidas como ações autorregulatórias, desenvolvidas a partir de padrões de comportamentos, associadas às características temperamentais, qualidade dos vínculos, e ao contexto em que o sujeito está inserido^(9,10,14-16)^.

A TMC organiza o *coping* em um modelo estrutural hierárquico de três níveis: 1) nível inferior (respostas de *coping*); 2) nível intermediário (estratégias de *coping*) e 3) nível superior (inclui 12 categorias/famílias de *coping:* “resolução de problemas”, “busca de informações”, “desamparo”, “fuga”, “autoconfiança”, “busca de apoio”, “delegação”, “isolamento”, “acomodação”, “negociação”, “submissão”, “oposição”. Cada uma dessas categorias/famílias de enfrentamento se relaciona a um tipo de avaliação cognitiva do evento estressor, uma necessidade básica e um processo adaptativo^(9,10)^.

A avaliação cognitiva do evento estressor pode ser classificada como um desafio, quando o indivíduo acredita que é possível lidar com o evento estressor ou como uma ameaça quando ele percebe a situação como um prejuízo psicológico^(10,11)^.

As necessidades básicas do modelo da TMC podem ser relacionadas a competência, vínculo e autonomia. A competência refere-se ao desejo humano de conquistar objetivos e ser efetivo nas interações sociais. A necessidade de estabelecer vínculos refere-se ao processo de manter relacionamentos interpessoais próximos e criar laços confiáveis para se sentir valorizado e a autonomia refere-se a liberdade para realizar escolhas nas interações com o ambiente^(9,10,14-16)^.

No modelo, são considerados três processos adaptativos definidos como interpretações individuais sobre a situação potencialmente estressora. O processo adaptativo autorreferencial, coordenação das ações e das contingências no ambiente está relacionado à necessidade psicológica de ter competência. O processo adaptativo coordenação da confiança e dos recursos sociais disponíveis está relacionado à necessidade de vínculo. O processo adaptativo coordenação das preferências e opções disponíveis para fazer escolhas relaciona-se à necessidade psicológica humana básica de autonomia^(9-11)^.

Em situações de fala em público, as estratégias de enfrentamento podem auxiliar o indivíduo a lidar melhor com a situação estressora^(1,3,17,18)^. Alguns exemplos dessas estratégias são, estudar o conteúdo do que será apresentado, treinar a fala em público para outras pessoas, buscar apoio com colegas ou investir em assessorias comunicativas^(1-3,17-19)^. Embora essas e outras estratégias sejam preconizadas pela literatura, não se encontrou instrumentos que autoavaliassem as estratégias de enfrentamento para fala em público^(20)^. Os instrumentos de autopercepção existentes são poucos, entre eles a *Self-Statements During Public Speaking – SSPS* ^(5)^ *que avalia a dimensão cognitiva da fala em público, a Public Speaking Anxiety Scale* – PSAS^(6)^ que mensura os componentes comportamentais, cognitivos e fisiológicos da ansiedade ao falar em público e a *Personal Report of Public Speaking Apprehension*p- PRPSA^(20)^ que avalia o nível de desconforto ou nervosismo que os indivíduos experimentam ao enfrentar situações de fala diante de uma audiência, incluindo o medo de ser julgado.

Desta forma, propusemos uma escala de *coping,* voltada para avaliação da fala em público: Escala *Coping* para Fala em Público (ECOFAP). A escala foi elaborada segundo o referencial teórico da Fala em Público e o referencial teórico da TMC^(9,10,14-16)^. Esse modelo teórico foi escolhido devido à abrangência da TMC para os mecanismos de autorregulação, uma vez que esse construto é influenciado por processos genéticos, fisiológicos e sociais específicos dos ciclos vitais.

O desenvolvimento da ECOFAP seguiu os passos metodológicos necessários à sua validade e robustez científica. Todas as etapas de validação e confiabilidade do instrumento foram seguidas, de acordo com o *Standards for Educational and Psychological Testing (SEPT),* manual que orienta a elaboração e validação de testes, e que preconiza a apresentação de cinco evidências de validade: conteúdo do teste; processos de resposta; estrutura interna; relação com outras variáveis e evidências baseadas nas consequências dos testes^(21)^.

Neste artigo, mostraremos as evidências de validade do conteúdo e processo de resposta^(22,23)^. A validade baseada no conteúdo está relacionada ao formato das questões/itens, incluindo estrutura sintática e semântica^(23-26)^ e os procedimentos necessários para pontuá-los^(25,26)^. O processo de resposta consiste na aplicação da versão do instrumento, reformulada, após consenso entre juízes especialistas, em diferentes estratos da população-alvo, para verificar o desempenho dos respondentes.

Diante do exposto, o objetivo deste estudo foi apresentar as evidências de validade do instrumento ECOFAP, baseadas no conteúdo e nos processos de resposta.

## Método

Estudo metodológico de elaboração e validação de instrumento. Seguiu-se o modelo de elaboração de instrumentos proposto por Luíz Pasquali^(24,25)^_,_ que contempla procedimentos teóricos, empíricos e analíticos, atendendo aos critérios de validade orientados pelo *Standards for Educational and Psychological Testing (SEPT)* ^(21)^. O estudo foi aprovado pelo Comitê de Ética em Pesquisa (CEP) sob o parecer número 5.735.670/2022.

O construto teórico da ECOFAP foi elaborado com base nos preceitos teóricos da fala em público e da TMC^(9,10,14-16)^. O processo de obtenção das evidências de validade baseadas no conteúdo foi realizado em duas etapas: 1) metodologia para a elaboração dos itens e 2) metodologia de avaliação de juízes especialistas. O processo de obtenção de evidências de validade baseadas nos processos de resposta foi realizado em uma única etapa intitulada: 1) Etapa de validação da resposta com indivíduos com e sem dificuldades de fala em público.

### Evidências de validade baseada no conteúdo

1. Primeira etapa: metodologia para a elaboração dos itens

- Fontes para elaboração dos itens:

- Revisão integrativa da literatura sobre os instrumentos de avaliação da fala em público por Marinho et al.^(20)^.
- Consolidado sobre o construto da fala em público, alicerçado na experiência clínica dos autores, profissionais com mais de 10 anos de experiência.
- Alinhamento do item de acordo com as bases conceituais do modelo estrutural da TMC^(9,10,14-16)^.
- Entrevistas com pessoas que apresentam dificuldades para falar em público em atendimento fonoaudiológico para aperfeiçoamento comunicativo em uma clínica-escola de uma universidade pública brasileira.
- Definição sintática e semântica dos itens, segundo os 10 critérios de Pasquali^(25)^.
  1. Critério comportamental: o item deve expressar um comportamento.
  2. Objetividade: facilidade na identificação da resposta.
  3. Simplicidade: o item deve expressar ideia única.
  4. Clareza: ser compreensível por todos os estratos da população alvo. Evitar frases longas
  5. Relevância: a frase/ expressão deve ser capaz de avaliar o construto em questão.
  6. Precisão: cada item deve ser distinto dos demais.
  7. Variedade: variar a linguagem utilizada, o uso dos mesmos termos confunde as frases e dificulta diferenciá-las, formular os itens, como metade na afirmativa e metade na negativa.
  8. Modalidade: não utilizar expressões extremas como “péssimo” e “excelente”.
  9. Tipicidade: frases com expressões típicas do atributo;
  10. Credibilidade: item não deve parecer incoerente, sem propósito ou inapropriado à faixa etária para a qual se destina.

A quantidade de itens do construto seguiu a orientação proposta por Pasquali^(25)^, que preconiza que um instrumento deve ter no mínimo vinte itens. Os verbos iniciais dos itens da ECOFAP foram formulados para representar uma ação/esforço. Cada uma das doze famílias de *coping* deveria ter pelo menos dois itens.

1. Segunda etapa: metodologia de avaliação dos juízes especialistas

A primeira versão da ECOFAP, elaborada com 46 itens, foi analisada por um comitê de juízas especialistas, composto por dez profissionais, cinco fonoaudiólogas e cinco psicólogas, seguindo as premissas do Método Delphi, que tem por objetivo reunir e sistematizar, por consenso, opiniões de especialistas sobre determinado tema^(26-28)^.

As juízas eram naturais de vários estados brasileiros. As juízas, fonoaudiólogas eram especialistas em voz, doutoras, com experiência clínica na área da voz profissional há mais de cinco anos. As cinco juízas psicólogas, eram doutoras e pesquisadoras experientes no construto *coping.*

O recrutamento dos juízes aconteceu, no período de dezembro de 2021 a junho de 2022, com o envio de uma carta convite, enviada por *email,* na qual estavam anexados o link do formulário Google Forms, o Termo de Consentimento Livre e Esclarecido TCLE, constando os objetivos do estudo e as orientações para análise do material com as instruções para o julgamento de cada item proposto pelos pesquisadores.

### Passos para avaliação das juízas

1. Avaliar a pertinência teórica do item, ou seja, se o conteúdo do item representava o construto que ele pretendia medir. O avaliador deveria marcar sua resposta em uma escala *Likert*, com pontuação de 3 para adequado, 2 para indeciso e 1 para inadequado.
2. Avaliar a pertinência textual do item, ou seja, se o item apresentava adequação semântica, vocabulário e expressava a ideia que se queria avaliar. O avaliador deveria marcar sua resposta em uma escala *Likert,* com pontuação de 3 para adequado, 2 para indeciso e 1 para inadequado.
3. Justificar a avaliação e propor modificações para cada item julgado como inadequado.
4. Classificar o item em uma das 12 famílias de *coping* da TMC: “resolução de problemas”, “busca de informações”, “desamparo”, “fuga”, “autoconfiança”, “busca de apoio”, “delegação”, “isolamento”, “acomodação”, “negociação”, “submissão”, “oposição”. Ressalta-se que os itens foram aleatorizados para evitar viés de seleção.
5. Indicar se o item poderia representar mais de uma família de *coping*, em caso positivo a juíza deveria informar qual família poderia representar.
6. Após todas as sugestões e comentários dos avaliadores, os pesquisadores realizaram as modificações necessárias e organizaram a segunda versão da ECOFAP, que novamente passou pela avaliação das juízas, seguindo os mesmos passos avaliativos. A segunda versão foi enviada com 60 itens.

Após a segunda avaliação do comitê de especialistas, a ECOFAP foi readequada de acordo com os comentários e analisada quantitativamente. Os itens da escala foram alocados em um banco de dados e foi calculado o Índice de Validade de Conteúdo por item (IVC-I) pelo número de juízas concordantes com o item dividido pelo número total de avaliadores. Foram aceitos apenas itens com IVC-I com valores > de 0,78, seguindo os preceitos teóricos de Polit et al.^(29)^ Essa análise foi realizada no programa estatístico SPSS versão 25.0. Pela análise realizada 18 itens foram excluídos originando uma terceira versão de 42 itens.

### Evidências de validade baseadas nos processos de resposta

Nesse processo de evidência verifica-se se há falhas na formulação e dificuldades de compreensão dos itens, e avalia-se se as questões do instrumento estão adequadas em vários estratos da população. Esse processo foi construído em uma única etapa:

1. Etapa de validação da resposta com indivíduos com e sem dificuldades de fala em público

A terceira versão da ECOFAP, com 42 itens, foi avaliada por uma amostra de conveniência, representada na Tabela 1. A amostra foi composta por 30 pessoas, entre 18 e 40 anos, frequentadores de uma universidade brasileira. A maioria dos participantes era do sexo feminino (66,6%), com ensino superior incompleto (53,2%), autorrelatou ter dificuldades para falar em público (73,3%). Em relação a profissão a maior parte era formada por universitários (43,3%), seguida de secretários e técnicos administrativos (24,7%), professores universitários (21,5%) e porteiros (10,7%).

**Tabela 1:** Características sociodemográficas e autorrelato de dificuldades para falar em público (n=30)

**Variáveis**	**N**	**%**
**Sexo**		
Feminino	20	66,6
Masculino	10	33,4
**Faixa etária**		
18-22 anos	3	10,2
23-26 anos	12	43,1
27-31 anos	9	26,4
32-40 anos	6	20,3
**Escolaridade**		
Ensino médio	9	26,6
Superior incompleto	18	53,2
Superior completo	6	20,2
**Autorrelato de dificuldades para falar em público**		
Sim	22	73,3
Não	8	26,7
**Profissão**		
Porteiro(a)	3	10,7
Estudantes universitários	12	43,1
Professores universitários	6	21,5
Secretários técnicos administrativos	9	24,7

Legenda: N = números de casos, % = frequência

A coleta aconteceu no mês de julho do ano de 2022. Foram incluídos na amostra indivíduos maiores de 18 anos, de ambos os sexos e alfabetizados. Todos os participantes assinaram o Termo de Consentimento Livre e Esclarecido TCLE, constando os objetivos do estudo e as orientações para o julgamento de cada item proposto pelos pesquisadores. Para controlar possíveis vieses, todas as entrevistas foram realizadas por dois voluntários fonoaudiólogos. Antes de aplicar o instrumento, os pesquisadores forneceram um treinamento para os fonoaudiólogos voluntários, explicando o objetivo dessa etapa de validação, e orientando quanto a detecção de dificuldades operacionais, como tempo de aplicação do instrumento, reações não verbais dos entrevistados (expressões faciais, dúvida, impaciência, manifestações de ansiedade e linguagem corporal) e sugestões dos participantes. Tais características deveriam ser registradas pelos fonoaudiólogos voluntários logo após aplicação de cada pergunta qualitativamente, sem utilização de escores.

Depois que cada pergunta foi respondida pelo participante, em escala *Likert* de 0 (discordo totalmente) a 5 (concordo totalmente), o avaliador questionou sobre a clareza do item, perguntando: “*Você entendeu a pergunta?”* e solicitou que o respondente repetisse a questão como ele tinha entendido *“Por favor, repita a pergunta do jeito que você entendeu”*. Quando a resposta tinha algum elemento diferente da proposta original, transcreveu-se a resposta no momento da entrevista. O uso dessa estratégia de paráfrase permitiu analisar a compreensão do que foi solicitado, possibilitando modificações.

Após a aplicação da ECOFAP, os pesquisadores se reuniram com os voluntários e avaliaram qualitativamente, cada item quanto à compreensão, reações não-verbais e aplicabilidade por parte dos respondentes classificando-os como adequados, quando não foi necessário realizar ajustes e inadequados, quando o item não foi compreendido de forma satisfatória. Os autores da ECOFAP fizeram as modificações que julgaram necessárias, em consenso, elaborando uma nova versão da ECOFAP.

Após a análise quali e quantitativa obteve-se a terceira versão da ECOFAP que os autores intitularam de versão piloto. A versão piloto foi formada por 48 itens e foi avaliada por indivíduos com e sem dificuldades de fala em público na etapa de validação baseadas nos processos de resposta.

## RESULTADOS

Os resultados apresentam as evidências de validade baseadas no conteúdo do teste e nos processos de resposta da ECOFAP.

No Quadro 1 visualiza-se 24 itens avaliativos propostos pelos pesquisadores, categorizados nas seis famílias de *coping* relacionadas à avaliação cognitiva da situação estressora como desafio, e os comentários do comitê de especialistas sobre cada item. Os itens 1,3,4,5,9,10, 16 foram considerados adequados pelo comitê de especialistas. Os itens 2,7,8,13,17 e 18 também foram considerados adequados, mas os juízes propuseram modificações sintáticas. Os itens 6,11,12,14,15,19-24 foram considerados inadequados, não representando uma estratégia de *coping.*

**Quadro 1:** Itens avaliativos 1ª versão ECOFAP, relacionados à avaliação cognitiva da fala em público como um desafio (n=24)

**Famílias de *coping***	**1ª versão ECOFAP**	**Comentários 1ª versão ECOFAP**
**Resolução de problemas**: tentativa ativa de modificar a situação ou suas consequências. Planejar estratégias, ações instrumentais.	1-Listo os principais tópicos do que vou falar	-
	2-Traço uma ligação entre as minhas ideias	Substituir “traço” por “faço”,”estabeleço
	3-Treino o que vou falar	-
	4-Planejo minha apresentação para falar melhor	-
	5-Procuro falar com clareza	-
	6- Mantenho uma voz firme	Item não representa uma estratégia de *coping*
**Busca de informações: t**entativa ativa para aprender mais sobre a situação. Ler, estudar, perguntar aos demais.	7-Busco aprender mais em assessorias de fala em público	O termo “assessorias” pode não ser popular para maioria das pessoas
	8-Participo de cursos de oratória	Participo como aluno
	9-Faço leituras para aprender mais sobre como falar em público	-
**Autoconfiança**: tentativa ativa de diminuir a angústia emocional. Regulação comportamental e autofala positiva.	10-Mantenho uma postura corporal confiante	-
	11-Penso que grande parte do meu nervosismo não é visível	Item não representa uma estratégia de *coping*
	12-Evito pensamentos negativos sobre mim	Item não representa uma estratégia de *coping*
	13-Procuro manter-me tranquilo.	Completar a frase com: “enquanto eu falo”
	14-Tento enxergar a oportunidade como um crescimento	Item não representa uma estratégia de *coping*
**Busca de apoio:** tentativa ativa de usar recursos sociais disponíveis para lidar com a situação estressora por meio de busca de contato, ajuda, referenciamento social.	15-Ouço a opinião dos meus colegas	Item não representa uma estratégia de *coping*
	16-Busco apoio profissional	-
	17-Ensaio para pessoas conhecidas	Completar “ensaio minha fala”
	18-Converso com colegas mais experientes	Reformular item
**Acomodação:** tentativa ativa de redirecionar a atenção para longe da situação estressora, ocupando-se com atividades prazerosas ou modificando a visão sobre a situação. Restruturação cognitiva, aceitação, minimização.	19-Tenho medo de falar, mas continuo	Item não representa uma estratégia de *coping*
	20-Sinto meu coração disparado, mas acredito que logo vai passar	Item não representa uma estratégia de *coping*
	21-Aprendo coisas boas com cada nova experiência	Item não representa uma estratégia de *coping*
**Negociação:** tentativa ativa de encontrar novas opções, elaborar uma solução de compromisso entre as prioridades individuais e as restrições da situação, estabelecer novos objetivos. Troca.	22-Estabeleço pequenas metas a cada dia	Item não representa uma estratégia de *coping*
	23-Preparo respostas antevendo perguntas	Item não representa uma estratégia de *coping*
	24-Troco minha preocupação pela ideia de que vou me sair bem	Item não representa uma estratégia de *coping*

Forma de apresentação adaptada de Vasconcelos e Nascimento^(30)^, Skinner e Zimmer-Gembeck^(16)^ e Skinner et al.^(9)^

No Quadro 2 visualiza-se os outros 22 itens avaliativos da primeira versão da ECOFAP, categorizados nas seis famílias de *coping* relacionadas a avaliação da fala em público com uma ameaça e os comentários do comitê de especialistas sobre cada item. As juízas avaliaram os itens 36,37,38,40 como adequados. Os itens avaliativos 25,27,41 também foram considerados adequados, mas sugeriram-se modificações. Os itens 26 a 35, 39, 42 a 46 foram considerados inadequados, não representando uma estratégia de *coping.*

**Quadro 2:** Itens avaliativos 1ª versão ECOFAP, relacionados à avaliação cognitiva da fala em público como ameaça (n=22)

**Famílias de *coping***	**1ª versão ECOFAP**	**Comentários 1ª versão ECOFAP**
**Desamparo**: O indivíduo se considera impotente frente à situação. Passividade, dúvida, desânimo.	25-Acho que vou esquecer as palavras	Substituir “acho” por “tenho medo de”
	26- Sinto que não consigo me acalmar	Item não representa uma estratégia de *coping*
	27- Travo na hora de falar	“fico paralisado, fico travado”
**Fuga:** tentativa ativa para libertar-se da situação e das dificuldades relacionadas a ela. Pessimismo, afastamento mental.	28-Falo mais rápido para ficar livre da situação	Especificar situação de falar em público
	29- Evito olhar para as pessoas enquanto falo	Item não representa uma estratégia de *coping*
	30- Desejo que minha fala termine logo	Item não representa uma estratégia de *coping*
	31- Acredito que não sou objetivo quando falo	Item não representa uma estratégia de *coping*
**Delegação:** o indivíduo tem pena de si ao avaliar que não tem recursos suficientes para lidar com o evento estressor. Reclamação, autoculpa, lamentação.	32-Considero difícil saber onde colocar as mãos enquanto falo	Item não representa uma estratégia de *coping*
	33-Acredito que ninguém presta atenção enquanto falo	Item não representa uma estratégia de *coping*
	34-Imagino que ninguém entende o que eu falo	Item não representa uma estratégia de *coping*
	35--Sinto que falar em público é um sofrimento	Item não representa uma estratégia de *coping*
**Isolamento:** tentativa ativa de evitar as pessoas ou impede que elas saibam sobre como se sente frente a situação. Afastamento social, evitação dos demais, solidão.	36-Evito lugares onde tenha que falar.	-
	37-Evito dar uma opinião, mesmo quando tenho algo a dizer	-
	38-Escondo dos colegas o que sinto quando sou obrigado a falar	-
	39- Desvio o olhar do ouvinte	Item não representa uma estratégia de *coping*
	40-Evito conversar com meus colegas sobre minha dificuldade de falar em público	-
**Submissão:** tentativa ativa de manter o foco passivo e repetitivo nos aspectos negativos e prejudiciais da situação. Rigidez, ruminação, pensamentos intrusivos.	41-Acredito que vou fracassar	Completar a ideia do item
	42-Penso que se eu errar não consigo retomar a minha fala	Item não representa uma estratégia de *coping*
	43-Penso que não vou saber responder a uma pergunta	Item não representa uma estratégia de *coping*
**Oposição:** tentativa ativa de usar comportamentos de raiva, explosão, para remover obstáculos impostos pela situação estressora. Agressão, explosão, culpar os outros.	44-Sinto que ninguém simpatiza comigo	Item não representa uma estratégia de *coping*
	45-Fico cansado de ter que repetir o que digo	Item não representa uma estratégia de *coping*
	46- Acredito que ninguém presta atenção enquanto falo	Item não representa uma estratégia de *coping*

Forma de apresentação adaptada de Vasconcelos e Nascimento^(30)^, Skinner e Zimmer-Gembeck^(16)^ e Skinner et al.^(9)^.

Nas Tabelas 2 e 3 visualiza-se a segunda versão da ECOFAP de 60 itens. Na Tabela 2 visualiza-se 30 itens reescritos após sugestões do comitê de juízes especialistas, classificando nas 6 famílias de *coping* relacionadas a avaliação da situação estressora como desafio, com os comentários da segunda rodada de avaliação e o índice de validade de conteúdo por item (I-ICV). Os itens 2, 3, 4, 6, 7, 9, 10, 11, 12, 18, 19, 21, 22, 23, 26, 27, 29 e 30 foram bem avaliados apresentando I-ICV representativos, e os itens avaliativos 1, 5, 8, 13, 16, 17, 20, 24, 25, 28 foram considerados inadequados e foram retirados.

**Tabela 2:** Itens avaliativos da 2ª versão da ECOFAP categorizado nas famílias de *coping* ligadas a avaliação como desafio, com comentários e índice de validade do conteúdo por item (n=30)

**Famílias de *coping***	**2ª versão ECOFAP**	**Comentários 2ª versão ECOFAP**	**I-ICV**
**Resolução de problemas**	1-Estudo o conteúdo sobre o que vou falar	Representa a família *de coping* busca de informações	0,64
	2-Organizo previamente as ideias	-	0,90*
	3-Treino o que vou falar em voz alta	-	0,90*
	4-Preparo uma apresentação visualmente atraente	-	0,85*
	5- Concentro-me no conteúdo da minha apresentação enquanto estou falando	Item não representa uma estratégia de *coping*	0,70
	6-Me esforço para pronunciar as palavras com clareza	-	0,89*
**Busca de informações**	7- Leio sobre como falar em público	-	0,90*
	8- Faço treinamentos sobre como falar em público	Representa outras famílias: busca de apoio, resolução de problemas	0,60
	9 Observo como bons falantes falam	-	0,82*
	10-Observo como colegas agem nessa situação	-	0,85*
	11- Assisto vídeos sobre como falar bem	-	0,89*
**Autoconfiança**	12-Tento manter uma postura corporal confiante	-	0,92*
	13-Digo a mim mesmo (a) que essa é uma oportunidade de aprendizado	Retirar item	0,30
	14-Concentro-me em pensamentos positivos	-	0,80*
	15- Procuro manter-me tranquilo enquanto falo	-	0,95*
	16-Tento enxergar a oportunidade como um crescimento	Item não representa uma estratégia de *coping*	0,50
**Busca de apoio**	17-Peço ajuda a colegas e amigos	Item não representa uma estratégia de *coping*	0,55
	18-Treino a minha fala com pessoas conhecidas	-	0,80*
	19-Busco apoio de um profissional	Completar com “para aprender a falar bem em público”	0,85*
	20-Ouço a opinião dos meus colegas	Item não representa uma estratégia de *coping*	0,60
	21-Pergunto a colegas mais experientes o que eles fazem para falar bem	-	0,80*
**Acomodação**	22-Faço caminhadas	Completar com “para me distrair antes de uma apresentação em público”	0,80*
	23-Assisto televisão	Completar “para desviar minhas preocupações com a minha apresentação”	0,95*
	24-Faço algum tipo de relaxamento ou meditação	Retirar item	0,20
	25-Faço exercícios respiratórios	Item não representa uma estratégia de *coping*	0,50
	26- Escuto música	Completar “para relaxar antes de falar em público”	0,80*
**Negociação**	27-Estabeleço pequenas metas a cada dia	Completar “com o objetivo de ser um bom falante”	0,90*
	28-Preparo respostas antevendo perguntas	Item não representa uma estratégia de *coping*	0,45
	29-Tento substituir a tarefa de falar por uma outra atribuição	-	0,80*
	30-Negocio um prazo maior para me preparar	-	0,95*

Legenda: I-ICV: Índice de validade de conteúdo para cada item da escala

* I-ICV>0,78

**Tabela 3:** Itens avaliativos 2ª versão ECOFAP categorizado nas famílias de *coping* ligadas a avaliação como ameaça, com comentários e índice de validade do conteúdo por item (n=30)

**Famílias de *coping***	**2ª versão ECOFAP**	**Comentários 2ª versão ECOFAP**	**I-ICV**
**Fuga**	31-Indico outra pessoa para falar em meu lugar	“Evito essa tarefa e indico outra pessoa para falar em meu lugar”	0,80*
	32- Adio a situação de falar em público	-	0,90*
	33- Acelero a fala para terminar logo	-	0,95*
	34-Invento uma desculpa para não falar em público	-	0,85*
	35-Mudo de assunto quando alguém me faz uma pergunta	Item não representa uma estratégia de *coping*	0,40
**Delegação**	36-Reclamo com colegas por ter que ser eu a falar	-	0,90*
	37-Me culpo por não querer falar	-	0,85*
	38-Reclamo com meus familiares por ter que passar por essa situação	Retirar item	0,60
	39-Fico lamentando que não consigo falar bem	Retirar item	0,55
	40-Espero que alguém assuma a situação por mim	-	0,85*
**Desamparo**	41-Fico desanimado ao pensar que vou ter que falar em público	Substituir Fico desanimado por “desanimo”	0,90*
	42-Travo na hora de falar em público	“Me imagino travado”	0,80*
	43-Antecipo resultados negativos	Item não representa uma estratégia de *coping*	0,40
	44-Tento falar melhor, mas não consigo	-	0,95*
	45- Reflito sobre o que falar, mas não encontro uma saída	-	0,80*
**Isolamento**	46-Desvio o olhar do ouvinte	Item não representa uma estratégia de *coping*	0,45
	47-Evito dar uma opinião, mesmo quando tenho algo a dizer	-	0,90*
	48Evito lugares onde tenha que falar	-	0,85*
	49- Evito conversar com meus colegas sobre minha dificuldade de falar em público	-	0,84*
	50-Escondo dos colegas o que sinto quando sou obrigado a falar	-	0,92*
**Submissão**	51- Fico preso na ideia de que não adianta me preparar	Substituir “fico preso” por “persevero”	0,85*
	52-Insisto na ideia de que não terei sucesso	-	0,90*
	53-Preparo-me para o pior	Item não representa uma estratégia de *coping*	0,30
	54-Repito para mim mesmo que é difícil enfrentar essa situação	-	0,85*
	55-Fico ruminando as sensações negativas que a situação me causa	Item não representa uma estratégia de *coping*	0,25
**Oposição**	56-Respondo de forma ríspida quando alguém me interrompe	-	0,84*
	57-Culpo os outros por ter que falar	-	0,90*
	58-Demonstro irritação quando alguém discorda do que eu estou expondo	-	0,85*
	59- Me posiciono de forma brusca	Item não representa uma estratégia de *coping*	0,35
	60-Fico impaciente quando os outros pedem para eu repetir o que falei	Substituir “fico impaciente” por “reajo com impaciência”	0,95*

Legenda: I-ICV: Índice de validade de conteúdo para cada item da escala

* I-ICV>0,78

Na Tabela 3 temos 30 itens restantes da segunda versão da ECOFAP reescritos após sugestões do comitê de juízes especialistas, classificados nas 6 famílias de *coping* relacionadas a avaliação da situação estressora como ameaça, com os comentários da segunda rodada de avaliação e o índice de validade de conteúdo por item (I-ICV). As juízas classificaram os itens 31 a 34, 36, 37, 41, 42, 44, 45, 47, 48, 49, 50, 51, 52, 54, 56, 57, 58 e 60 como adequados e apresentaram I-ICV representativos. Os itens avaliativos 35, 38, 39, 43, 46, 53, 55 e 59 foram considerados inadequados, com baixo I-ICV.

O Quadro 3 apresenta a versão piloto da ECOFAP com 48 itens. Essa versão foi elaborada a partir da reestruturação da segunda versão da ECOFAP e aplicação do instrumento na amostra de conveniência de 30 pessoas, descrita no método desse artigo para obter evidências de validade baseadas nos processos de resposta.

**Quadro 3:** Itens avaliativos da versão piloto da ECOFAP nas famílias de *coping* (n=48)

**Famílias de coping**	**Versão piloto ECOFAP**
**DESAFIO**	
**Resolução de problemas**	1- Organizo previamente as ideias
	2- Treino o que vou falar em voz alta
	3-Preparo uma apresentação visualmente atraente
	4-Me esforço para pronunciar as palavras com clareza
**Busca de informações**	5-Leio sobre como falar bem em público
	6- Observo como bons falantes falam
	7-Assisto vídeos sobre como falar bem
	8- Observo como colegas agem nessa situação
**Autoconfiança**	9- Visualizo-me falando bem
	10-Tento manter uma postura corporal confiante
	11-Procuro manter-me tranquilo enquanto falo
	12-Concentro-me em pensamentos positivos
**Busca de apoio**	13- Busco apoio de um profissional para aprender a falar bem em público
	14- Pergunto a colegas mais experientes o que eles fazem para falar bem
	15- Treino a minha fala com pessoas conhecidas
	16- Procuro sugestões de como os colegas lidam com a situação
**Acomodação**	17- Escuto música para relaxar antes de falar em público
	18- Faço caminhadas para me distrair antes de uma apresentação em público
	19- Assisto televisão para desviar minhas preocupações com a minha apresentação
	20- Concentro-me em alguma coisa boa que possa vir dessa situação
**Negociação**	21- Estabeleço pequenas metas a cada dia com o objetivo de ser um bom falante
	22- Negocio um prazo maior para me preparar
	23-Esclareço para todos que não me saio bem nessa atividade
	24-Tento substituir a tarefa de falar por uma outra atribuição
**AMEAÇA**	
**Desamparo**	25-Desanimo ao pensar que vou falar em público
	26-Me imagino travado na hora de falar em público
	27- Tento falar melhor, mas não consigo
	28- Reflito sobre o que vou falar, mas não encontro uma saída
**Fuga**	29- Adio a situação de falar em público
	30- Invento uma desculpa para não falar em público
	31- Acelero a fala para terminar logo
	32- Evito essa atividade e indico outra pessoa para falar em meu lugar
**Delegação**	33- Reclamo com colegas por ter que ser eu a falar
	34- Me culpo por não querer falar em público
	35- Peço que alguém fale em meu lugar
	36- Me recrimino por não saber falar bem
**Isolamento**	37- Evito conversar com meus colegas sobre minha dificuldade
	38- Escondo dos colegas o que sinto quando sou obrigado a falar
	39- Evito lugares onde tenha que falar
	40- Evito dar uma opinião em público mesmo quando tenho algo a dizer
**Submissão**	41- Persevero na ideia de que não adianta me preparar
	42-Insisto na ideia de que não terei sucesso
	43-Repito para mim mesmo que é difícil enfrentar essa situação
	44- Digo a mim mesmo que a minha apresentação vai dar errado
**Oposição**	45- Respondo de forma ríspida quando alguém me interrompe
	46- Demonstro irritação quando alguém discorda do que eu estou falando
	47-Reajo com impaciência quando os outros pedem para repetir o que falei
	48- Culpo os outros por ter que falar

Após aplicação os pesquisadores, em consenso, avaliaram cada questão, categorizando como adequadas as que não precisaram fazer ajustes e inadequadas as questões que não foram compreendidas de maneira satisfatória. O item “*Leio sobre como falar em público”* foi reescrito *“Leio sobre como falar bem em público”.* Os itens *“Visualizo-me falando bem”,* “*Procuro sugestões de como os colegas lidam com a situação”, “Concentro-me em alguma coisa boa que possa vir dessa situação”, “Esclareço para todos que não me saio bem nessa atividade”, “Peço que alguém fale em meu lugar” e “Digo a mim mesmo que minha apresentação vai dar errado”* foram acrescentados como opinião dos participantes.

## DISCUSSÃO

Os resultados mostraram as evidências de validade baseadas no conteúdo e nos processos de resposta da ECOFAP. Estas evidências foram primordiais para ajustar os aspectos teóricos, contextuais, semânticos e sintáticos das versões iniciais da ECOFAP. Autores recomendam que a apresentação das etapas de validade baseadas no conteúdo e nos processos de resposta sejam divulgadas antes de se prosseguir para investigação das outras etapas de evidência de validade do instrumento, o que confere o rigor metodológico necessário à construção de instrumentos^(21)^.

A ECOFAP é uma escala de autoavaliação das estratégias de enfrentamento para fala em público. Sabe-se que instrumentos de autoavaliação, auxiliam o sujeito a se questionar sobre determinado aspecto, muitas vezes não relatados espontaneamente e influenciam na prontidão para mudança em relação ao seu problema^(31,32)^. A partir da autoavaliação também é possível verificar a percepção que a pessoa tem sobre a sua capacidade de autorregulação^(33)^_._ Autores discutem que a autorregulação é fundamental no processo de aprendizagem e generalização de novas habilidades, além de ser importante para manejo ou manutenção de comportamentos adquiridos a longo prazo^(34)^_._

A elaboração da ECOFAP se baseou na TMC, de Skinner et al.^(9,10,14-16)^, que compreende o *coping,* como uma ação autorregulatória, que monitora o comportamento de resposta a situações estressoras^(9,10,14-16,30)^. Outros estudos, da área da saúde, também utilizaram a teoria TMC para fundamentar o desenvolvimento de instrumentos de coping^(35,36)^.

A versão I da ECOFAP, foi elaborada com 46 itens, divididos entre as famílias e os seus respectivos processos adaptativos, como apresentados nos Quadros 1 e 2. Os itens foram aleatorizados para avaliação dos juízes, evitando o viés de análise^(36)^. Esse cuidado metodológico é realizado para que não haja uma tendência de o juiz avaliar todos os itens da mesma forma, sem considerar suas diferenças de conteúdo ou formato. Ao aleatorizar a ordem dos itens, cada item é avaliado de forma independente, proporcionando uma análise mais precisa e confiável, evitando-se também a fadiga do avaliador, pois não há um padrão repetitivo na apresentação dos itens^(36)^. Na avaliação qualitativa dos itens, observa-se que a maioria das sugestões foram em relação ao uso da linguagem, verbos e tipo de família de *coping.* Sabe-se que a linguagem do item precisa ser clara, e os aspectos sintáticos e semânticos da frase devem favorecer coesão e representar apenas uma ideia, compreensível, e direcionada à população alvo^(23-25)^.

Nesta vertente, alguns termos precisaram ser revisados, como por exemplo, substituir o termo “assessorias” por treinamentos ou cursos. Observa-se que 20 itens da I versão foram avaliados como inadequados, por não representarem estratégias de *coping* para lidar com a fala em público e sim um sentimento. Os verbos desses itens precisaram ser revistos para se configurarem como uma ação regulatória para se adaptar e enfrentar situações estressoras.

Estudos descrevem as dificuldades na elaboração dos itens de um instrumento e na classificação dos itens dentro das 12 famílias de *coping* da TMC^(37)^_._ Sabe-se que o processo de elaboração dos itens é um processo árduo, de rigor metodológico e que muitas vezes, depende do olhar de um especialista de fora do processo, o que reforça a importância dessa fase de validação do conteúdo. Após os comentários e sugestões na I versão, os autores se reuniram, discutiram e realizam um novo ajuste. Itens foram reformulados, ou retirados e outros foram incluídos e também reclassificados em outras famílias. Todas essas modificações só foram possíveis devido aos comentários dos especialistas, o que impulsionou os autores a reestruturar os itens, assim como observado em outros estudos na literatura^(37,38)^.

Na versão II da ECOFAP de 60 itens (Tabelas 2 e 3) observa-se uma maior aceitação dos juízes, com pequenas sugestões. Nesta fase se realizou uma avaliação quali e quantitativa. A avaliação quantitativa é recomendada para embasar a tomada de decisão dos pesquisadores quanto aos ajustes do instrumento, caracterizando a concordância entre os juízes através da análise de índice de validade de conteúdo^(24-26)^. Os resultados mostram que a grande maioria dos itens apresentou índice de validade de conteúdo (I-ICV) com alto grau de concordância, o que mostra que os itens estavam bem redigidos e seguiram adequadamente as etapas de validação para esse fim.

Na versão III da ECOFAP (Quadro 3), os 48 itens foram definidos a partir do resultado da análise do índice de validade de conteúdo, seguido de aplicação de estudo piloto em estratos diversificados da população-alvo e opiniões dessa população. Estudos apontam que investigar as evidências de validade baseadas no processo de resposta complementam as evidências de validade baseadas no conteúdo do teste, sendo altamente relevante para realizar ajustes no instrumento, baseados em reações verbais e não verbais da população alvo^(23,38)^. Nesta etapa foram sugeridos acréscimos de 5 itens e um item foi reescrito. Consideramos pertinentes as mudanças para cumprir os critérios de simplicidade propostos por Pasquali, adequando a linguagem a população-alvo^(24,25)^.

As limitações desse estudo estão relacionadas a ausência de estudo de validade de critério (comparação com medida externa), validade de construto (análise da estrutura fatorial), fidedignidade (estabilidade temporal por meio de teste-reteste e consistência interna) e estabelecimento de ponto de corte com significado clínico.

Ressaltamos que a primeira, segunda e a versão piloto aqui apresentadas não são a versão final da ECOFAP. A escala ECOFAP ainda passará pela etapa de investigação de evidências da estrutura interna, analisando-se as características psicométricas do instrumento. Uma vez concluído o processo de validação da ECOFAP, ela poderá ser aplicada nas assessorias e pesquisas fonoaudiológicas como instrumento de autopercepção e autorregulação da fala em público. Acredita-se que a escala poderá auxiliar a condução das assessorias e como medidas de desfecho nas pesquisas científicas.

## CONCLUSÃO

A ECOFAP é uma escala de autoavaliação das estratégias de enfrentamento para fala em público elaborada e obtida pelas evidências de validade baseadas no conteúdo do teste e nos processos de resposta. Apresenta itens com estruturas semântica e sintática adequadas e que representam o construto teórico de autorregulação da fala em público. Ressalta-se a importância dessas evidências serem consideradas por pesquisadores para utilizarem instrumentos confiáveis e apropriados para uma população alvo.
